# Isolation and adaptation of bovine herpes virus Type 1 in embryonated chicken eggs and in Madin–Darby bovine kidney cell line

**DOI:** 10.14202/vetworld.2016.222-225

**Published:** 2016-02-27

**Authors:** Devprabha Samrath, Sanjay Shakya, Nidhi Rawat, Varsha Rani Gilhare, Fateh Singh

**Affiliations:** 1 Department of Veterinary Microbiology, College of Veterinary Science and Animal Husbandry, Anjora Durg, Chhattisgarh, India; 2 Department of Veterinary Public Health, College of Veterinary Science and Animal Husbandry, Anjora Durg, Chhattisgarh, India; 3 Division of Animal Health, ICAR-Central Sheep and Wool Research Institute, Avikanagar-304501, Rajasthan, India

**Keywords:** Bovine herpes virus Type 1, chorioallantoic membrane, cytopathic effect, Madin–Darby bovine kidney cell line, pock

## Abstract

**Aim:**

Objective of the present study was to isolate bovine herpes virus Type 1 (BHV-1) from semen of infected bull and to adapt it onto embryonated eggs and Madin–Darby bovine kidney (MDBK) cell line. Further, the virus was identified by agar gel immunodiffusion (AGID) test.

**Materials and Methods:**

Semen samples were collected from five BHV-1 positive bulls previously confirmed for the presence of antibodies against BHV-1 using avidin-biotin enzyme linked immunosorbent assay test. The virus from semen samples was adapted in chorioallantoic membrane (CAM) of 11-day-old embryonated chickens eggs and in MDBK cell line. The presence of BHV-1 in infected CAM and cell culture fluid was confirmed by AGID test.

**Results:**

Virus infected CAM showed edema, congestion and thickening at first passage level. Small foci ranged from 1 to 2 mm in diameter, scattered all over the membrane were observed at first passage. More severe changes were observed in CAM after serial passaging. The large pock lesions, round in shape with opaque raised edge and depressed gray central area of necrosis ranged from 3 to 5 mm in diameter were developed at fourth passage. Blind passages in MDBK cell culture were made. The MDBK cell line at second passage level showed characteristic cytopathic effect *viz*. rounding of cells with shrinkage, followed by aggregation or clumping of cells which progressed rapidly and appeared as “bunch of grapes” at 72 h post inoculation. Few cells become elongated when compared with uninfected controls. A homogenate of CAM with distinct pock lesions and infected cell culture fluid developed precipitation line within 48 h against specific anti-BHV-1 immune serum by AGID test.

**Conclusion:**

BHV-1 was easily adapted in CAM of chicken embryos and in MDBK cell line. Virus infected CAM and cell culture fluid showed precipitin band by AGID test.

## Introduction

Bovine herpes virus Type 1 (BHV-1), an important pathogen of cattle, reported worldwide [1] comes under genus *Varicellovirus, Alphaherpesvirinae* subfamily of *Herpesviridae* family [2]. The viruses are enveloped have icosahedral nucleocapsid which is composed of 162 capsomers. The genome of the virus is linear and made up of double-stranded DNA 125 to 290 kbp in size [3]. The virus causes enormous economic losses to livestock industry by decreasing milk production and abortion. The virus causes a variety of clinical symptoms including rhinotracheitis, balanoposthitis, vulvovaginitis, abortion, and encephalitis [4]. Moreover in cattle, infection causes conjunctivitis, acute gastroenteritis, mastitis and repeat breeding [5,6]. In general, cattle above 6 months of age are affected by BHV-1 when maternal immunity has declined [7]. The BHV-1 infection occurs during direct contact between animals via respiratory, ocular or genital secretions. Frozen semen from infected bulls and contaminated equipment are another potential source of infection [8].

The virus is excreted through nasal and ocular secretion, semen and aborted placenta. Subsequent an acute infection, the virus get latent in the sensory ganglia of the animal [9]. BHV-1 induces immune suppression in cattle [10] which leads to secondary bacterial infections; as a result BHV-1 is an important cofactor in the bovine respiratory disease complex with immense financial impacts [2,11]. Infectious bovine rhinotracheitis (IBR) is classified in the list B of diseases by the Office International des Epizooties (OIE) [11].

BHV-1 can be readily isolated in cell culture of primary or secondary bovine kidney, lungs, testis, turbinate bone, trachea and established cell lines such as Madin–Darby bovine kidney (MDBK) cell line [12]. The virus produces cytopathic effect (CPE) in infected cells. Embryonated chicken eggs are commonly used for isolation of animal viruses due to its several advantages, adaptation of virus in embryonated eggs may be useful for large scale production of virus required for vaccine manufacturing. In the embryonated chicken eggs, BHV-1 produces pock lesion when inoculated via chorioallantoic membrane (CAM) route. In present investigation, isolation and adaptation of BHV-1 in CAM of developing chicken embryos and MDBK cell line have been carried out.

## Material and Methods

### Ethical approval

All the procedures have been conducted accordance with the approval from Institutional Animal Ethics Committee.

### Samples collection

A total of 464 serum samples were screened for presence of antibody against BHV-1 by using avidin-biotin enzyme linked immunosorbent assay (A-B ELISA) as per the standard protocol outlined in the user's manual supplied with kit was procured from Project Directorate on Animal Disease Monitoring and Surveillance, Hebbal, Bengaluru, India. Out of 422 cattle sera 158 were found positive, and 3 sera out of 42 were found positive in buffaloes. Fresh semen sample was collected from five infected bulls (2 cattle and 3 buffaloes) showing high titer of antibody against BHV-1. Samples were collected by per rectal massage of ampullae and seminal vesicle [13]. All samples were collected in the capped sterile plastic vials and transported on ice to the laboratory and processed as the method by Madbouly *et al*. [14] as quickly as possible, after freezing and thawing several times (3-5 cycles) semen was diluted in ratio of 1:10 with fetal bovine serum (OIE) then centrifuged at 3000 rpm for 10 min. The suspension of these specimens was filtered through 0.45 μm size filters and used for virus isolation.

### Adaptation of BHV-1 onto CAM of chicken embryos

For virus isolation, 11-day-old embryonated chickens eggs were inoculated through CAM route [14]. Five serial passages were performed for obtaining the good titer of virus and the presence of BHV-1 was confirmed by AGID test [15].

### Adaptation of BHV-1 onto MDBK cell line

The BHV-1 was adapted in MDBK cell line, following the procedure described by the OIE [16]. The presence of BHV-1 in cell culture fluid was confirmed by AGID test [15]. Infected and corresponding control coverslips were fixed in methanol and stained with May–Grunwald Giemsa stain (MGG) [17] for microscopic examination using inverted microscope.

## Results

### A-B ELISA test

BHV-1 antibodies in serum were assessed by A-B ELISA.

### Adaptation of BHV-1 in chicken embryos

**Figure-1:**
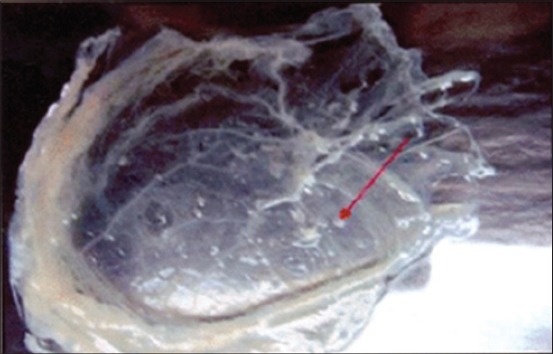
Chorioallantoic membrane showing 1-2 mm pocks at second passage level.

**Figure-2:**
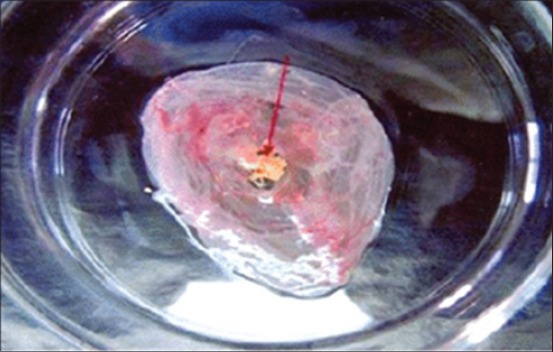
Chorioallantoic membrane showing large size pock at fourth passage level.

In the present investigation, the semen from BHV-1 infected bulls was inoculated in chicken embryos via CAM. The CAM showed edema, congestion and thickening at first passage level. Small foci ranged from 1 to 2 mm in diameter scattered all over the membrane were observed at the second passage (Figure-1).The large pock lesion rounded in shape with opaque raised edge and depressed gray central area of necrosis ranged from 3 to 5 mm in diameter were developed at the fourth passage (Figure-2). The result of present study clearly indicated large and small pock lesions on the CAM. More severe changes were observed in CAM after serial passaging which showed adaptation of BHV-1 via CAM route. However, mortality was not seen in embryo during the study.

### Isolation of BHV-1 in MDBK cell culture

**Figure-3:**
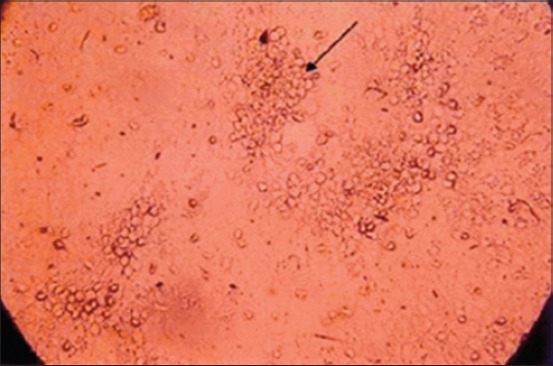
Madin–Darby bovine kidney cell line infected with bovine herpes virus type 1 showing severe rounding at 72 h post inoculation.

**Figure-4:**
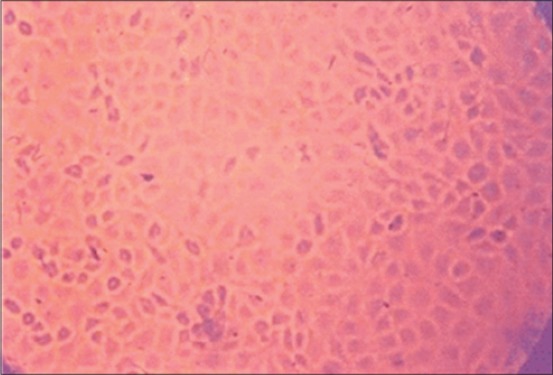
Uninfected monolayer of Madin–Darby bovine kidney cell line.

Blind passage in MDBK cell culture was made to attempt virus isolation. The MDBK cell line at second passage level showed characteristic CPE *viz*. rounding of cells with shrinkage, followed by aggregation or clumping of cells which progressed rapidly and appeared as “bunch of grapes” at 72 h post inoculation (PI) (Figure-3). Few cells become elongated when compared with uninfected controls (Figure-4).

### Detection of BHV-1 antigen

Homogenate of CAM with distinct pock lesions and infected cell culture fluid was tested to identify the presence of virus by AGID test using specific anti-BHV-1 immune serum developed precipitation line within 48 h.

## Discussion

Five semen samples from BHV-1 suspected bulls (2 cattle and 3 buffaloes) were collected and assessed using A-B ELISA test and antibody against BHV-1 was found positive in the samples. Seropositivity for IBR antibodies highlighted the circulation of virus among the livestock population of Odisha [18]. At initial passage level infected CAM become thick, edematous and congested. Typical pocks were observed onto CAM on subsequent passages. The characteristics pock lesions were also observed by Madbouly *et al.*, Madbouly and Hussein [14,19] when they collected semen sample from dairy herds having genital form of infection. The result of present study showed large and small pock lesions were on CAM. More severe changes were observed in CAM after serial passages, indicating adaptation of BHV-1 *via* CAM route.

The BHV-1 infected and control monolayers were stained with MGG stain, and the CPE were examined microscopically. CPE was developed on second passage level which includes rounding, aggregation and syncytia formation. The result of present study are in accordance to the finding of Deka *et al*. [20] who observed characteristic rounding and clumping of cells followed by degeneration and detachment of the MDBK cell monolayer at 72-96 h PI. CPE changes began to appear at the fourth passage on the 2^nd^ day PI was reported by Mahmoud *et al*. [21]. MDBK cell cultures have been used for adaptation and cultivation of BHV-1 by several other workers [22-24]. The propagation of BHV-1 in continuous cell lines is advantageous because it avoids the problems associated with the use of primary cell cultures. The quality of continuous cell lines is more stable than primary cell cultures. Primary cell cultures, however, must be derived each time from specific pathogen free sources to assure their freedom from interfering antibodies. In the present study, the MDBK cell line was used because of its bovine kidney origin and availability. BHV-1 produced CPE in MDBK cell lines at second passage level, indicated the possibility of adaptation of virus. More serial passages will be required for better adaptation of virus with consistent CPE.

## Conclusions

The virus produced pocks on CAM of chicken embryos and characteristic CPE was observed in MDBK cell line. Homogenate of CAM and infected cell culture fluid developed precipitation line within 48 h by agar gel immunodiffusion.

## Authors’ Contributions

DS carried out the experiment and drafted the final manuscript. SS designed the experiment. NR, VRG and FS helped in the sample collection and analysis. All authors read and approved the final manuscript.
